# The Dilemma of Choice for Duchenne Patients Eligible for Exon 51 Skipping The European Experience

**DOI:** 10.3233/JND-221648

**Published:** 2023-05-02

**Authors:** Annemieke Aartsma-Rus, Liesbeth De Waele, Saskia Houwen-Opstal, Janbernd Kirschner, Yvonne D. Krom, Eugenio Mercuri, Erik H. Niks, Volker Straub, Hermine A. van Duyvenvoorde, Elizabeth Vroom

**Affiliations:** a Leiden University Medical Center, Leiden, the Netherlands; b Duchenne Center Netherlands, the Netherlands; c Department of Paediatrics, University Hospitals Leuven, Leuven, Belgium; d Department of Development and Regeneration, KU Leuven, Leuven, Belgium; e Department of Rehabilitation, Donders Institute for Brain, Cognition and Behaviour, Radboud University Medical Center, Amalia Children’s Hospital, Nijmegen, the Netherlands; f Department of Neuropediatrics and Muscle Disorders, Medical Center – University of Freiburg, Faculty of Medicine, Freiburg, Germany; g Department of Pediatric Neurology, Catholic University, Rome, Italy; h Centro Clinico Nemo, Fondazione Policlinico Agostino Gemelli IRCCS, Rome Italy; i John Walton Muscular Dystrophy Research Center, Newcastle University, Newcastle upon Tyne, United Kingdom; j Duchenne Parent Project

**Keywords:** Duchenne muscular dystrophy, dystrophin, exon 51 skipping, antisense oligonucleotide, clinical trial

## Abstract

Antisense oligonucleotide (ASO) mediated exon skipping aims to reframe dystrophin transcripts for patients with Duchenne muscular dystrophy (DMD). Currently 4 ASOs have been approved by the Food and Drug Administration targeting exon 45, 51 and 53 based on low level dystrophin restoration. Additional studies to confirm functional effects are ongoing. Furthermore, efforts are ongoing to increase muscle specific delivery of ASOs. Consequently, there are 5 clinical trials ongoing or planned for exon 51 skipping ASOs in Europe. While exon 51 skipping applies to the largest group of patients, DMD expert centers do not have sufficient numbers of patients or capacity to run all these trials in parallel. Even at a national level numbers may be too scarce. At the same time, some families now face the choice between participation in different clinical trials of exon 51 skipping, sometimes in addition to the choice of participating in a micro-dystrophin gene therapy trial. In this opinion paper, we outline the challenges, compare the different exon 51 skipping trials, and outline how different European centers and countries try to cope with running multiple trials in parallel for a small group of eligible patients.

## INTRODUCTION

**Fig. 1:**
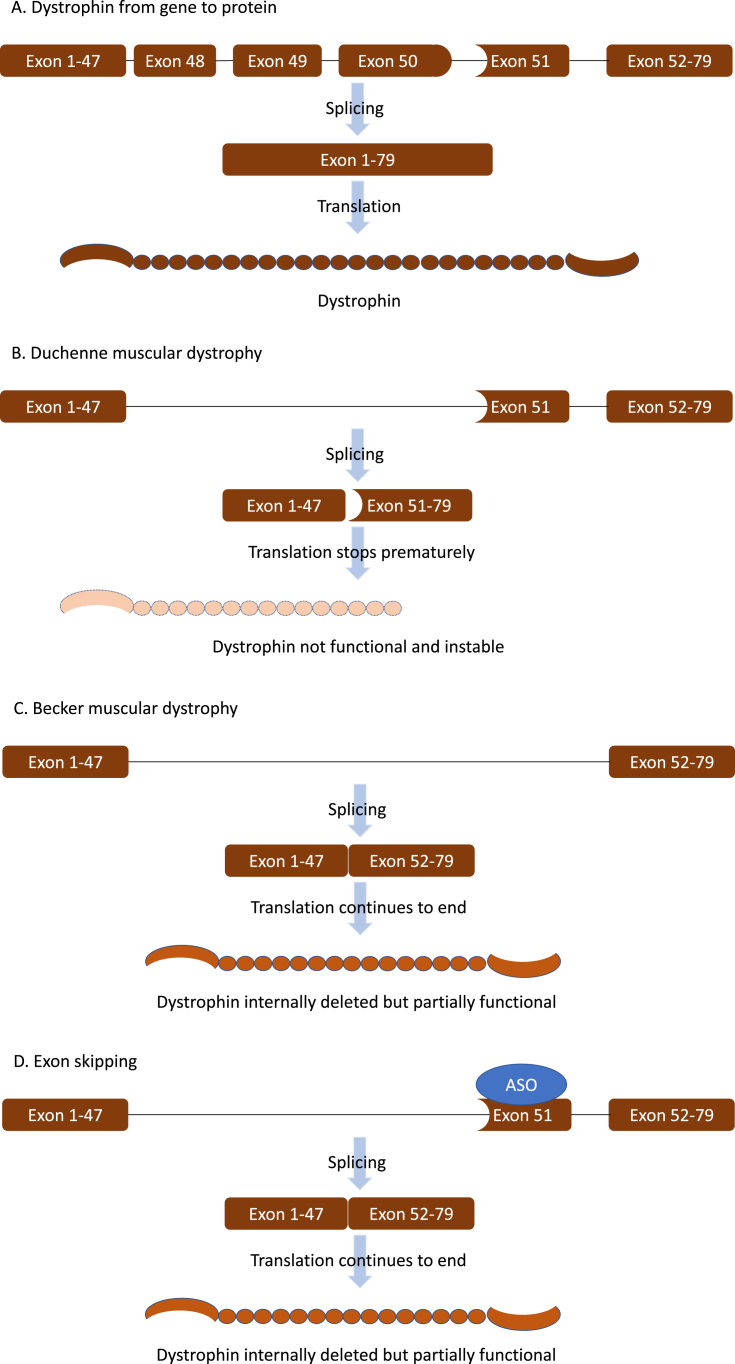
The principle of exon skipping. A. The DMD gene contains the code for the dystrophin protein. The code is distributed over 79 exons. During pre-mRNA splicing the exons will be joined together to form the mRNA that is translated into dystrophin protein. B. In Duchenne muscular dystrophy (DMD) the reading frame is disrupted by a mutation (in this example by a deletion of exon 48-50, the shape of exon 47 does not match that of exon 51). This causes protein translation to stop prematurely resulting in a non-functional, instable dystrophin protein. C. In Becker muscular dystrophy (BMD) mutations maintain the open reading frame. This allows production of a partially functional dystrophin. D. Exon skipping uses antisense oligonucleotides (ASO) that target a specific exon, to hide it from the splicing machinery. The exon is not included into the mRNA, which restores the reading frame allowing patients with DMD to produce partially functional proteins as in BMD.

Duchenne muscular dystrophy (DMD) is caused by loss of function mutations in the *DMD* gene that prevent the production of full-length dystrophin (Fig. 1AB) [1]. Normally dystrophin acts as a shock absorber during muscle fiber contraction by connecting F-actin in the cytoskeleton to the dystrophin-glycoprotein complex and further to the extracellular matrix surrounding each muscle fiber. Lacking dystrophin, skeletal muscle fibers of patients with DMD are continuously damaged, eventually resulting in replacement of muscle tissue by adipose and fibrotic tissue. This is accompanied by progressive loss of muscle function. Most patients lose ambulation before the age of 12, require assisted ventilation around the age of 20 and die in the 2nd–4th decade of life due to respiratory or heart failure [1].

The dystrophin domains crucial for protein function are located at the N- and C-terminal end and are interspaced by 24 spectrin-like repeats and 4 hinge regions. Interestingly, mutations in the *DMD* gene that do not disrupt the reading frame and allow production of internally deleted dystrophins are associated with progressive Becker muscular dystrophy (BMD) (Fig. 1 C) [2]. These patients have a later onset of symptoms and a slower disease progression, confirming that the internally deleted dystrophins are partially functional.

Exon skipping therapies are based on the fact that generally out-of-frame mutations in the *DMD* gene lead to DMD while in-frame mutations lead to BMD (Fig. 1D). The exon skip approach uses antisense oligonucleotides (ASOs), short pieces of chemically modified RNA that specifically target an exon in the pre-mRNA. Upon binding of the ASOs, the exon is hidden from the splicing machinery and skipped from the mature mRNA. This enlarges the deletion to an in-frame deletion, which reframes the transcript, thus allowing production of internally deleted, partially functional dystrophin proteins as produced by BMD patients [3].

Exon skipping is a mutation-specific approach. Depending on the location of the mutation, different exons need to be skipped to restore the reading frame. However, because about two thirds of patients with DMD carry a deletion of one or more exons and 75% of these cluster in a hotspot between exon 42 and exon 55, skipping certain exons applies to larger groups of patients [3, 4]. The exon with the largest applicability is exon 51, skipping of which applies to 14% of patients. Other exons applying to larger groups are exon 45 (an additional 9% of patients), exon 53 (an additional 8% of patients), and exon 44 (an additional 6% of patients). Beyond that group sizes drop rapidly to 4% (exon 50 skipping), 3% (exon 43 skipping) and 2% (exon 8 skipping) [4]. For now, each ASO has to go through all phases of clinical development separately. To allow inclusion of sufficient patients, initial development has focused on exon 51, 45 and 53 skipping.

Efficient ASOs were identified in control and patient-derived cell cultures. However, preclinical *in vivo* studies were done in the *mdx* mouse model using ASOs targeting mouse exon 23. Systemic treatment resulted in dystrophin levels of up to 100% depending on the chemical modifications and doses used [5, 6]. In patients with DMD, however, efficiencies were much lower. Currently four ASOs have been approved by the USA Food and Drug Administration (FDA): eteplirsen (exon 51 skipping), viltolarsen (exon 53 skipping), golodirsen (exon 53 skipping), and casimersen (exon 45 skipping) [1, 7]. These approvals were based on restoration of dystrophin levels in muscle biopsies at levels of<1% (eteplirsen), 1% (golodirsen and casimersen), and 5% (viltolarsen). The sponsors have been requested by the FDA to provide confirmatory evidence that these levels of dystrophin slow down disease progression. None of these ASOs have been approved in Europe. Marketing authorization was sought for eteplirsen with the European Medicine Agency (EMA), but this was denied due to the paucity of data, with a limited number of patients [12] and not having a placebo arm [8]. No applications have been done for the other ASOs.

While it is currently not clear whether the approved ASOs can slow down disease progression for DMD, there is consensus that if they do, there is room for improvement: restoring dystrophin at higher levels is anticipated to have a larger clinical impact. The main cause for the low efficiency is thought to be poor delivery to skeletal and heart muscle.

Multiple companies have worked on ways to improve exon skipping efficiency by improving delivery to muscle, with a strong focus on improving exon 51 skipping ASOs. Currently there are five clinical trials planned and ongoing for exon 51 skipping in European centers (Tables 1 and 2 and supplementary figure). In addition, there are also gene therapy clinical trials ongoing that aim to deliver a micro-dystrophin gene to skeletal muscle and heart muscle using adeno associated viral vectors (AAV) [9]. This means that families eligible for exon 51 skipping now face the choice of participating in one of five potential exon 51 skipping trials and sometimes also micro-dystrophin gene therapy trials on top of a plethora of clinical trials that aim to improve muscle quality and that are not mutation specific. At the same time, clinicians face the challenge of running multiple trials in parallel for a small cohort of eligible participants and properly advising families on the pros and cons of each. In addition, it is impossible to properly power phase 3 trials to confirm clinical benefit for each of the separate exon 51 skipping compounds as there probably are not enough eligible patients to populate all these clinical trials.

**Table 1:** Trial Characteristics

Compound	Sponsor	Clinicaltrial.gov ID	Phase	N	Duration (weeks)	Hospital visits	Dosing	Placebo?	Biopsy?	Age-range	OLE?	Sibling protocol?	CAB?
Eteplirsen	Sarepta Therapeutics	NCT03992430	3	154	144	49	Weekly IV	No	2	4–13 years (A)	yes	?	Yes
Vesleteplirsen	Sarepta Therapeutics	NCT04004065	2	60	104	56	Monthly IV	No	2	7–21 years (A and NA)	yes	?	Yes
PGN-EDO51	Pepgen	None yet	1–2	?	?	?	Monthly IV?	?	?	?	?	?	Yes
Dyne-251	Dyne Therapeutics	NCT05524883	1–2	46	134	38	Monthly IV	Yes, first 24 weeks	2	4–16 years (A)	yes	?	Yes
BMN351	BioMarin	None yet	1–2	?	?	?	?	?	?	?	?	?	Yes

A ambulant, NA non ambulant, IV intravenous.

**Table 2:** Preclinical and clinical information

Compound	Backbone and conjugates	Preclinical efficiency	Preclinical safety	Dystrophin expression	Clinical safety	Comments
Eteplirsen	PMO	Exon 23 PMO induces dystrophin restoration in *mdx* mice (higher dose, more dystrophin)	Well tolerated	0.4% dystrophin restoration after 48 weeks treatment at 30 mg/kg and 50 mg/kg	Well tolerated	New trial tests higher doses (30, 100 and 200 mg/kg)
		Eteplirsen not tested in Duchenne mouse models
Vesleteplirsen	PMO with R rich peptide conjugate (pPMO)	Exon 23 pPMO induces more dystrophin restoration than PMO in *mdx* mice.	Well tolerated	Up to 2% dystrophin after 3 monthly doses of 30 mg/kg	Hypomagnesemia observed	Clinical trial put on hold twice by FDA
		Vesleteplirsen not tested in Duchenne mouse models, Vesleteplirsen induced exon 51 skipping induced in non human primates
PGN-EDO51	PMO with R rich peptide conjugate	Exon 23 pPMO induces more dystrophin restoration than PMO in mdx mice.	Hypomagnesemia observed in non human primates	Not yet tested	Hypomagnesemia observed in healthy volunteers
		PGN-EDO51 not tested in Duchenne mouse models, PGN-EDO51 induced exon 51 skipping induced in non human primates
Dyne-251	PMO with transferrin targeting ab	Exon 23 abPMO induces dystrophin restoration in *mdx* mice.	Well tolerated in non human primates	Not yet tested	Not yet tested
		Dyne-251 not tested in Duchenne mouse models. Dyne-251 induces exon 51 skipping in non human primates
BMN351	2OMePS, other modifications?	BMN351 induced 40% dystrophin restoration in humanized mouse model.	Well tolerated in mice	Not yet tested	Not yet tested

2OMePS 2’-O-methyl RNA with phosphorothioate backbone, Ab antibody, PMO phosphorodiamidate morpholino oligomer, R arginine.

In this opinion paper, we outline the challenges, compare the different exon 51 skipping trials, and provide different solutions for the neuromuscular field on how to cope with running multiple trials in parallel for a small group of eligible patients.

## OVERVIEW OF PLANNED AND ONGOING TRIALS

An overview of the five different exon 51 ASOs currently or soon to be tested in clinical trials is given in Table 1 (characteristics of clinical trials) and Table 2 (information on ASOs, preclinical and clinical safety and dystrophin restoration).

The MI51ION trial from sponsor Sarepta Therapeutics (NCT03992430) assesses the safety and efficiency of higher doses of eteplirsen, the first ASO to be approved by the FDA in 2016 at a dose of 30 mg/kg bodyweight per week. Eteplirsen is a phosphorodiamidate morpholino oligomer (PMO), which is a third generation ASO chemistry that is uncharged and resistant to nuclease degradation. Eteplirsen is delivered with weekly intravenous infusions. Bioavailability is limited, and most of the ASO will be filtered out by the kidney shortly after the infusion. Tolerability of eteplirsen is generally very good. Reported side effects include balance disorders, vomiting, skin rash, bruising, joint pain, upper respiratory tract infection and catheter pain (https://www.rxlist.com/exondys-51-side-effects-drug-center.htm). However, the efficiency of eteplirsen is low with dystrophin restoration levels measured at 0.3% after 48 weeks of treatment and 0.9% after 188 weeks of treatment [10]. The MI51ION trial is a two part clinical trial, consisting of a short dose-escalation phase assessing the safety of two higher doses of eteplirsen (100 and 200 mg/kg/week) in a limited number of patients [10] followed by a randomized trial comparing the highest tolerated dose versus the approved 30 mg/kg/week for 144 weeks with an recruitment target of 144 ambulant patients. Dystrophin levels will be compared in biopsies obtained at baseline and either 24, 48 or 144 weeks of treatment. The primary endpoint is the North Star Ambulatory Assessment (NSAA). As such only ambulant patients, aged 3–14 who require exon 51 skipping to restore dystrophin production are eligible to participate.

The MOMENTUM trial (NCT04004065) from sponsor Sarepta Therapeutics assesses the safety and efficiency of vesleteplirsen. This is the PMO eteplirsen linked to an arginine-rich peptide (pPMO), which is known to increase delivery of compounds in tissues in general. As such, the conjugate should result in higher ASO uptake in skeletal muscle and therefore higher levels of exon skipping and dystrophin restoration. However, it is known that arginine-rich peptides can result in renal toxicity [11].

In the *mdx* mouse model for DMD, pPMOs targeting exon 23 induced levels of exon 23 skipping and murine dystrophin restoration of over 50% after a single injection of 80 mg/kg [12]. Furthermore, treatment of non-human primates with vesleteplirsen, which hybridizes to both human and monkey exon 51, resulted in exon 51 skipping in healthy muscle. Due to the improved delivery efficiency, the pPMO can be injected monthly rather than weekly. The vesleteplirsen clinical trial also occurs in two stages, of which the dose finding stage has been completed already. This revealed that a dose of 30 mg/kg monthly for 3 months resulted in an ∼2% increase of dystrophin [13]. In stage B, patients are being treated with 30 mg/kg/months vesleteplirsen for up to 2 years in an open label set up. The primary endpoint is the change in dystrophin levels from baseline to 28 weeks of treatment. Ambulant and non-ambulant patients aged 7–21 years and requiring exon 51 skipping to restore dystrophin expression can participate. The estimated enrollment is 60 patients. While the compound was generally well tolerated, hypomagnesemia was reported in multiple patients resulting in a temporary trial hold imposed by the FDA. Magnesium supplementation is used to counteract the low magnesium levels in serum. The underlying cause for the hypomagnesemia is unknown. It could potentially be a marker for kidney damage resulting in magnesium reabsorption problems.

PGN-EDO51, developed by Pepgen, is also a pPMO, but with a different arginine-rich peptide. During presentations at patient advocacy meetings, the company has presented preclinical studies, where conjugation of the peptide to a PMO showed increased efficiency in skipping mouse exon 23 and restoring dystrophin in the *mdx* mouse when compared to unconjugated PMO. Furthermore, PGN-EDO51 resulted in exon 51 skipping in muscles from non-human primates and healthy volunteers, but hypomagnesemia was observed as well. The company has announced the planning of a clinical trial in patients with DMD. However, details of the trial design have not been published.

Dyne-251, developed by Dyne Therapeutics, is a PMO linked to an antibody fragment (FAB) targeting the transferrin receptor, which is highly expressed on skeletal muscle. In the *mdx* mouse, treatment with an exon 23 FAB-PMO results in targeted delivery of the PMO to the muscle and increased levels of exon skipping and dystrophin restoration [14]. Treatment with Dyne-251 resulted in exon 51 skipping in non-human primates. Dyne Therapeutics has initiated a clinical trial (NCT05524883) that will last for 104 weeks, with a placebo group for the first 24 weeks. Patients will be treated with monthly doses of Dyne-251 via intravenous infusion. Dystrophin restoration will be assessed at week 25. The trial will include ambulant patients with DMD requiring exon 51 skipping for dystrophin restoration aged 4–16 years. Recruitment is targeted at 46 patients.

Finally, BMN351 is developed by BioMarin, the sponsor previously involved in developing drisapersen, an exon 51 targeting 2’-O-methyl phosphorothioate ASO, which has been tested in hundreds of DMD patients in phase 1, 2 and 3 clinical trials. The compound failed to reach the primary endpoint in a phase 3 clinical trial [10]. Notably, the phosphorothioate backbone allows lower ASO doses to be used as it prevents renal clearance and increases ASO bioavailability. However, the backbone also can induce side effects. Subcutaneous delivery of drisapersen was associated with severe, long-term injection site reactions in most patients and resulted in thrombocytopenia for 2% of patients [15]. BMN351 is a newly developed ASO that is based on the same chemical modifications as drisapersen, but also contains additional, as yet undisclosed, modifications.

Biomarin plans on initiating its first in human clinical trial with BMN351, an exon 51 targeting ASO developed using optimized PS chemistry targeting a novel, upstream, splice enhancer site demonstrating enhanced exon skipping and dystrophin production in preclinical. The company has presented at patient advocacy meetings how the compound was tested in a humanized mouse model (hDMDdel52/*mdx*), which allows preclinical mouse studies using human exon 51 targeting ASOs [16]. Preclinical data in this mouse model with functional impairment on the motorater scale, showed restored expression of near-full-length dystrophin protein to levels that converted the phenotype from rapid functional loss to durable preservation of strength and ambulation. Regulatory filing is expected in early 2023 to enable initiation of the clinical phase of development. The outline of the clinical trial is currently not publicly disclosed.

It should be mentioned that the preclinical studies are often done in the *mdx* mouse model with an ASO targeting exon 23 of the mouse Dmd gene transcript. This makes extrapolation to the human situation more difficult, because the efficiency of the mouse exon 23 ASO may be different from the human exon 51 ASO and the functionality of a dystrophin lacking amino acids in the beginning of the protein encoded by exon 23 will differ from a dystrophin lacking amino acids from the hot spot deletion region [17]. Biomarin by contrast, used a humanized animal model (hDMDdel52/*mdx*) and therefore the preclinical results in the mouse were obtained with the same compound that will be used in the clinical trial. However, obviously a mouse is not a human and whether the compound has similar efficiency and safety remains to be tested in the clinical trial.

From a safety perspective, Dyne-251 may induce an immune response to the FAB-arm, while BMN351 treatment may be associated with phosphorothioate backbone related toxicity such as thrombocytopenia, and an inflammatory response. However, the occurrence and seriousness of these responses varies for different ASOs, so this is something that will remain to be assessed in the clinical trials.

## HOW TO CHOOSE, THE FAMILY PERSPECTIVE

It is clear that most of the trials target 4–14 year old ambulant patients. Gene therapy trials sponsored by Pfizer, Sarepta/Roche, Solid Bio, and Genethon aiming to deliver a micro-dystrophin are ongoing as well and also target this age range. A limited group of patients and their families now has a choice of which trial they want to participate in. We will refrain from indicating which option is the best choice, as this will vary based on the personal preferences and situation of each family. However, when making the decision, several aspects will play a role. The trial burden is significant for all of the protocols and a major limitation for families to participate, although it varies for different trials, with a duration ranging from 104 weeks (veseteplirsen) to 144 weeks (high dose eteplirsen), and dosing regimen of monthly (vesleteplirsen, PGN-EDO51, Dyne 251) or weekly/fortnightly intravenous injections (eteplirsen). All trials involve two biopsies except for BMN351 and PGN351 for which this is not yet known. Notably, while DMD families understand the need for muscle biopsies, they also have indicated biopsies are a major burden [18]. In addition to the trial burden, families also will consider whether the trial involves a placebo arm (Dyne-251 during first stage of the trial), whether the patients will be enrolled in an open label extension trial after they have completed the trial (available for all studies except PGN351 and BMN351 for which it is not yet disclosed). Patients and families also want to know whether there is a sibling program allowing open label treatment of affected siblings that do not fulfill the inclusion criteria. Whether a sibling program is currently not known for any of the compounds. Finally, families likely want to know if the sponsors involved the patient community in the trial design. For these compounds all companies discussed their plans with the Duchenne community advisory board (CAB).

In addition to the burden, families will base their decision on available preclinical and clinical data with regards to efficiency and risk. Only vesleteplirsen and eteplirsen have been used in DMD patients so far, revealing that eteplirsen has a good safety record, but limited efficiency, while vesleteplirsen has higher efficiency but is also associated with side effects such as the mentioned hypomagnesemia. Also, the risk of unknown side effects is higher for vesleteplirsen, as this is tested for the first time, while patients have been treated with eteplirsen for over 5 years. Based on the similarity of veleteplirsen and PGN-EDO51, a similar efficiency/risk profile can be expected for PGN-EDO51.

As mentioned, families may also have the option to enroll in a gene therapy trial provided that they do not have antibodies against the AAV vector used (AAV74, AAV8 or AAV9). The gene therapy treatment involves a single infusion. However, follow up visits are planned for 5 years or more to establish long term safety and efficacy. From the ∼100 patients treated so far it is clear that AAV can achieve delivery of the micro-dystrophin genes to skeletal muscle resulting in expression levels of up to 74% in up to 80% of muscle fibers [9]. However, as has been disclosed at patient advocacy meetings by the companies involved, there are risks involved with gene therapy infusions with reported serious side effects including transient kidney failure, liver damage, transient liver failure, sepsis, myositis, myocarditis, rhabdomyolysis, and in one case death. Notably, these severe side effects are not experienced by all patients, but they can occur. In addition, for a subset of patients with deletions removing the N-terminal part of the protein, an immune response to the micro-dystrophin was observed that resulted in rhabdomyolysis and myocarditis. Consequently, patients with deletions in the start of the gene have been excluded from participation in micro-dystrophin trials.

Finally, it is not known yet how functional the micro-dystrophin is and what the duration of micro-dystrophin expression will be. The micro-dystrophin is significantly shorter than the smallest dystrophin reported for a BMD patient. As the micro-dystrophin is only partially functional, with time patients will lose transgenes due to muscle turnover and there will be dilution of transgenes due to muscle growth. While gene therapy is often seen as a ‘one time fix’, this is not the case for DMD. Exon 51 skipping might result in more functional dystrophin. However, currently the expression levels of micro-dystrophin are much higher than those achieved after exon skipping. Furthermore, patients that have been treated with gene therapy are currently excluded from participation in any other clinical trials.

## HOW TO ORCHESTRATE THIS –THE CLINICIAN PERSPECTIVE

While some compounds may appear more attractive based on preclinical data, there are caveats, such as the fact that for some compounds dystrophin restoration was achieved with mouse exon 23 ASOs. Furthermore, *mdx* mice have a much less severe pathology than humans and therefore there will be more target pre-mRNA transcripts available than in patients. As such, we believe it is important to facilitate clinical testing of the different compounds rather than prioritizing selected ASOs based on preclinical data. The combined targeted recruitment for the four protocols that are currently in the public domain is 260 DMD patients requiring exon 51 skipping. Some of the protocols do not involve a pivotal phase 3 design, which will increase the total number of required patients in future protocols even more. Given that each clinical trial site has a limited number of patients who are eligible for the exon 51 skipping trials, it will be challenging if not impossible to run all 5 trials at one site or even within one country, as this will likely lead to some trials not including any patients at that site. One also has to consider that setting up a clinical trial with many trial sites that individually recruit only very few or single patients is associated with high administrative burden and costs and might also jeopardize trial standardization and quality.

In Belgium three sites are conducting clinical trials for patients with DMD without aligning as yet. However, trial sites are running out of patients and likely future trials will only be run in one or two sites.

In Germany five sites are already conducting clinical trials or planning to do so. The German patient registry for DMD is helpful to identify eligible patients, but nevertheless numbers are limited at individual centers. In addition, long travel distances often impede participation due to the need for regular infusions. Home treatment is not easy to implement within the context of a clinical trial in Germany.

In Italy an academic network for DMD has a central dataset including all DMD patients followed in 14 tertiary care centers that also provide information on genotype and functional status. Each time there is a new study the academic network interacts with the Italian Parent Project who also have a large patient driven registry that does not completely overlap with the academic one. The combination of the two registries allows to reach a large number of DMD boys and adults so that all the patients in the two large registries who have the right appropriate criteria is informed of the option/s and can contact the clinical trial sites if interested or for further discussion.

In the Netherlands, two national referral centers that participate in the Duchenne Center Netherlands, have aligned with each other and the sponsors to set up trials in one of the sites with mutual referral of potential candidates. Recruitment and prescreening however, is done on a national level via the national registry, the Dutch Dystrophinopathy Database, and an active DMD patient organization (Duchenne Parent Project), the muscle disease patient organization (Spierziekten Nederland), and by approaching treating physicians through the database of the Laboratory for Diagnostic Genome analysis of the Leiden University Medical Center. Families are offered all available options with the caveat that certain trials are conducted outside of the site they visit for multidisciplinary care. While this is a solution to increase the number of options, this only works up to a number of trials that either center can run simultaneously.

Potential patient enrollment also depends on the number of available patients. Based on the number of boys born between January 1, 2008 and December 31, 2017 in the Netherlands (∼900,000), the incidence of DMD (1 in 5000 newborn males) and the percentage of exon 51 skippable patients (14%), an expected 25 eligible patients would be expected in the age range of 4–14 in the Netherlands. However, with extensive prescreening as described above, only 8 eligible patients were identified so far. This discrepancy can be due to improved prenatal diagnosis and a lower incidence of DMD in the Netherlands, natural variability in exon 51 skippable patients and/or because some patients are not in the diagnostic registry. The latter is less likely as DMD gene mutation analysis is centralized in one laboratory in the Netherlands. Even based on a theoretic 25 eligible patients on a population of 17.5 million inhabitants, one can extrapolate that to recruit 260 patients, a population of at least 182 million would be needed. Obviously, not all theoretically eligible patients are interested in participating in clinical trials, which further adds to the challenges of recruiting sufficient patients.

Another solution to facilitate recruitment is the example of the DMD Hub that was established in the UK to increase clinical trial capacity. The DMD Hub is a network of trial sites with trained staff which are funded to carry out clinical research studies and trials for DMD. It was set up and funded by the advocacy group Duchenne UK (https://www.duchenneuk.org/) as a collaboration with the neuromuscular centres of excellence in Newcastle and London. The DMD Hub provides a central resource offering advice, guidance and training to sites on setting up and running studies.

The DMD Hub is not only bringing research opportunities to the UK and funding the infrastructure needed to carry out trials, but working towards fairer and more effective patient recruitment, so that more people living with DMD have the chance to take part in research. In 2022, the DMD Hub launched a pilot project to assess the potential of a centrally-coordinated patient database to support trial recruitment. The Central Recruitment Project gathers information about people diagnosed with DMD and their preferences for taking part in research, so that eligible patients can be contacted by trial sites, regardless of their location in the UK.

While these solutions improve efficiency of conducting trials, it remains a challenge that there are not sufficient patients to confirm drug efficacy for each of the exon 51 skipping compounds, unless trial sites are established in countries with large patient numbers like India or China. This would on the other hand require the implementation of international care guidelines for DMD in these countries.

## FORWARD LOOK

In order to run clinical trials efficiently and to facilitate rapid recruitment more coordinative efforts are needed. National and international neuromuscular networks and patient organizations with associated patient registries (e.g. TREAT-NMD) can help to identify eligible patients in different countries. Feasibility databases such as the Care and Trial Site Registry can provide information about potential trial sites [19, 20]. Ideally, trial sponsors should also communicate and coordinate their activities, so that they do not compete for patients in some countries while in other countries no trial sites are opened.

For those working in the DMD field for decades, big advances have been made, with many clinical trials ongoing and DMD-specific drugs approved in Europe and the USA. However, it is clear that there is room for improvement and especially for the mutation-specific approaches this poses problems due to the limited number of patients available for trial participation. We have outlined the five different clinical trials that are ongoing or planned for the near future for exon 51 skipping. While we cannot recommend a specific compound, we do hope this opinion paper will help clinicians explain the pros and cons and different characteristics of each of the options, and to help families make the best choice for their individual situation. Furthermore, we hope our paper provides guidance for other clinical centers that are likely facing the same challenges we faced. Towards the future we anticipate that this challenge will increase, with even more exon 51 skipping ASOs, and similar situations for exon 53, exon 45 and exon 44 ASOs. While it may be idealistic, we hope that companies developing ASOs will align between themselves and focus on different exons, ideally those without an approved alternative or an ongoing clinical trial. While these subgroups are smaller than the ones requiring exon 51, 53, 45 and 44 skipping, there is no alternative for these patients yet and eligible patients would be recruited in a single trial, rather than being split over multiple trials for competing ASOs, that individually lack the power to show treatment efficacy.

## Supplementary Material

Supplementary Material
