# Supplementary material for: The Dilemma of Choice for Duchenne Patients Eligible for Exon 51 Skipping The European Experience
Source: J Neuromuscul Dis. 2023 May 2;10(3):315–25. doi: 10.3233/JND-221648 (PMC10200189; doi:10.3233/JND-221648)
Supplement: Supplementary Material [file jnd-10-jnd221648-s001.pptx]

## Slide 1
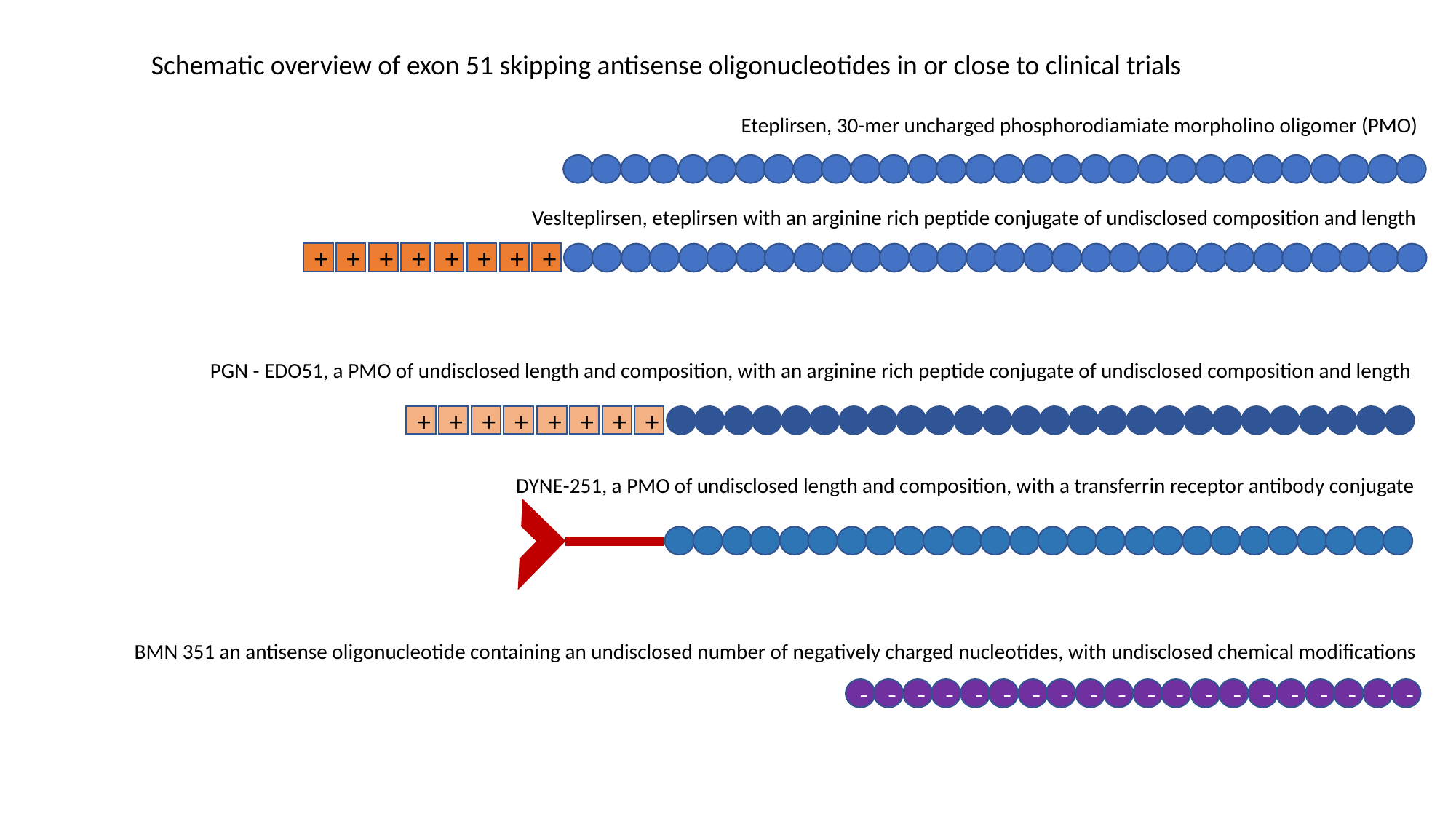

Schematic overview of exon 51 skipping antisense oligonucleotides in or close to clinical trials
Eteplirsen, 30-mer uncharged phosphorodiamiate morpholino oligomer (PMO)
Veslteplirsen, eteplirsen with an arginine rich peptide conjugate of undisclosed composition and length
+
+
+
+
+
+
+
+
PGN - EDO51, a PMO of undisclosed length and composition, with an arginine rich peptide conjugate of undisclosed composition and length
+
+
+
+
+
+
+
+
DYNE-251, a PMO of undisclosed length and composition, with a transferrin receptor antibody conjugate
BMN 351 an antisense oligonucleotide containing an undisclosed number of negatively charged nucleotides, with undisclosed chemical modifications
-
-
-
-
-
-
-
-
-
-
-
-
-
-
-
-
-
-
-
-
